# Manipulative Surgery

**Published:** 1917-04-07

**Authors:** Montagu Lomax


### THE EDITOR'S LETTER-BOX.

### Manipulative Surgery.

*To the Editor of* The Hospital.

Sir,—As a medical man who has himself benefited by Mr. Barker's skill, after having consulted orthodox surgeons in vain, I write to protest in the strongest fashion against the tone of your leading article in The Hospital. Of reasoned argument against the claims of Mr. Barker as an exponent of manipulative surgery, or the position which he has won in spite of the long-continued hostility and misrepresentation of the champions of medical orthodoxy, there is absolutely no trace, unless sneers and insinuations, both totally unjustified, may be considered arguments. You object that no record of Mr. Barker's failures is obtainable. I have yet to hear that records of the failures of orthodox surgeons are obtainable either. There would be some interesting and instructive reading if there were. No doubt Mr. Barker has had his failures; he would not be human if he had not. But since the greater number of the patients who apply to him represent the admitted failures of orthodox surgeons, and that in most cases Mr. Barker's methods have turned these "failures" into genuine, if unorthodox, "successes," the objection recoils with very appropriate force upon those who urge it. *De te fabula narratur*, Mr. Barker might with justice retort.

Again, your suggestion that violent measures must *of necessity* lead to injury in very many cases is met by the fact that the "violence" required (always supposing it exists) apparently does not in Mr. Barker's hands have this result, or surely out of the hundreds of cases annually treated by him we should long ago have had evidence to this effect. But so far as I am aware no one has yet written to the medical journals complaining that he has suffered at Mr. Barker's hands. The evidence has been all the other way.

In conclusion, it may not be without interest to recall what Mr. Walter Whitehead, president of the British Medical Association in 1902, had to say about the attitude which you, Sir, and those who think with you, take up on this question. Mr. Whitehead wrote: "I am convinced that the attitude adopted by the medical world towards the methods of manipulative surgery is only adding another regrettable page to those chapters in its history which it recalls with profound shame. Blinded by professional prejudice, the medical world has stolidly opposed every innovation and discovery which has been submitted to it." May I commend these words to you, Sir?—I am, yours, etc.,

Montagu Lomax, M.R.C.S., L.R.C.P.

---

F. William Axham, ex-M.R.C.S., L.R.C.P.E., concludes a letter too long for publication:—

"Sir Arbuthnot Lane has publicly pointed out that the failure of the profession, in regard to manipulative surgery, is due to a lack of knowledge of the methods used by the bonesetter. In that confession, Sir, lies the truth, the whole truth, and nothing but the truth, and I heartily commend it to your notice."

